# Bioactive isopimarane and 3,4-*seco* isopimarane diterpenoids from *Isodon amethystoides*

**DOI:** 10.1186/s13065-022-00880-4

**Published:** 2022-11-14

**Authors:** Chenliang Zhao, Lang Zhou, Wenjian Xie, Lihan Zhao, Chiyuan Zhang, Kang He, Jianghai Ye, Jingjie Zhang, Lutai Pan, Juan Zou, Hongjie Zhang

**Affiliations:** 1 College of Pharmacy, Guizhou University of Traditional Chinese Medicine, 4 Dongqing Road, Guiyang, Guizhou 550025 People’s Republic of China; 2 School of Chinese Medicine, Hong Kong Baptist University, 7 Baptist University Road, Kowloon Tong, Kowloon, Hong Kong, SAR People’s Republic of China

**Keywords:** *Isodon amethystoides*, Lamiaceae, Isopimarane diterpenoids, Cytotoxicity, Antibacterial

## Abstract

*Isodon amethystoides* (Lamiaceae) is a popular plant in folk medicine in the southern provinces of China. Our phytochemical investigation of the twigs and leaves of this plant led to the discovery of five new diterpenoids with isopimarane and 3,4-*seco* isopimarane scaffolds [isoamethinols A–E (**1**–**5**)], along with the known compound 3,4-*seco* isopimara-4(18),7,15-triene-3-oic acid methylester (**6**). The chemical structures of these compounds, including the absolute configurations of the new diterpenoids, were determined by comprehensive spectroscopic analyses and single crystal X-ray diffraction measurements. These compounds were evaluated for their biological activities against a panel of human cancer cell lines, gram-positive bacterial strains and HIV. Notably, the 3,4-*seco*-isopimarane isoamethinol D (**4**) showed toxicity to the cervical Hela cancer (Hela) cells with an IC_50_ value of 27.21 μM and the lung (A549) cancer cells with an IC_50_ value of 21.47 μM. Compound **4** also exhibited mild antimicrobial activity against the oral bacterial strain *Streptococcus mutans*. These findings suggested that the diterpenoids with a 3,4-*seco*-isopimarane diterpenoids isolated from *I. amethystoides* could provide a novel structure scaffold for the discovery of anticancer and antimicrobial compounds.

**Graphical Abstract:**

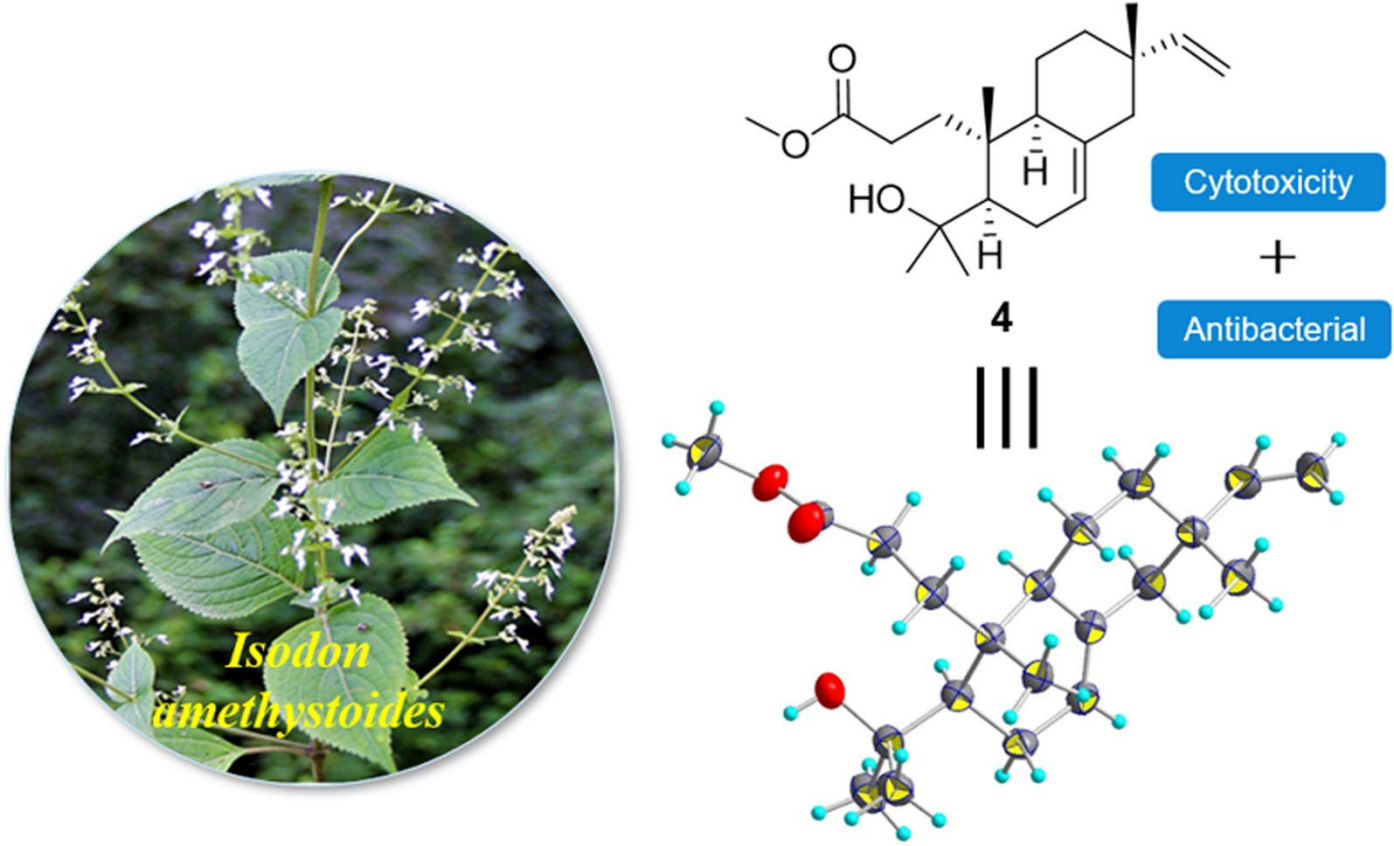

**Supplementary Information:**

The online version contains supplementary material available at 10.1186/s13065-022-00880-4.

## Introduction

Plants are known to produce abundant small molecules of secondary metabolites with novel and unique chemical structures and a wide range of bioactivities, thus providing an important source for the discovery and development of new drugs.

The plant genus *Isodon* (Lamiaceae), mainly distributed in the tropical and subtropical regions of Asia, has been increasingly studied in the recent years due to its structurally diverse constituents with medical applications [1–3]. As popular Chinese folk medicines, some species in the genus *Isodon* have been traditionally used for the treatment of gastritis, arthritis, hepatitis, cholecystitis, enteritis, and amenorrhea in the southern provinces of China [4–8]. Diterpenoids are a major class of natural compounds that are abundantly found in *Isodon* plants. They have been discovered to have a broad-spectrum of pharmacological activities, including anticancer, antiviral, and antibacterial properties [1–3, 5, 7].

**Fig. 1:**
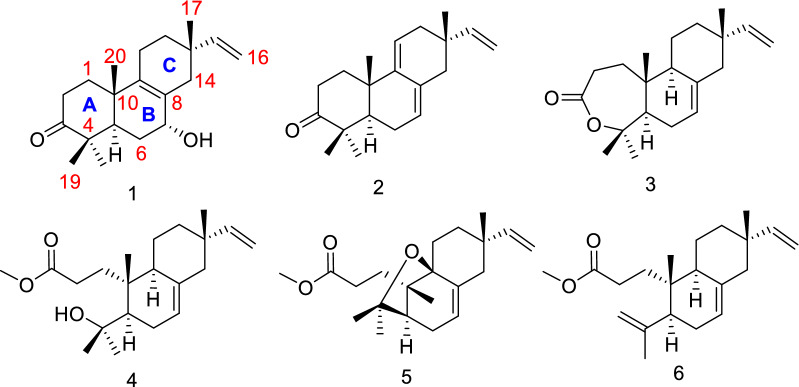
The chemical structures of compounds **1**–**6**

*Isodon amethystoides* is a medicinal plant belonging to the genus *Isodon*. Its leaf extract has been patented as a commercial drug with the trade-name “Wei Fu Chun Pian” for the treatment of precancerous lesions of gastric cancer lesions and also serves as an adjuvant therapy after gastric cancer surgery, as recorded in the Chinese Pharmacopoeia 2020 edition [9]. Previous phytochemical investigations of this species revealed significant differences in the chemical profiles of the secondary metabolites among the plants collected from various geographical areas [4–6]. Our recent research on a sample of this species collected from Libo County in Guizhou Province also led to the identification of a novel bioactive diterpene with 6/5/7 carbon skeleton, which showed inhibitory activity against the autoimmune disease-associated RORγt [10]. These prior findings encouraged us to carry out further phytochemical studies to discover additional new bioactive diterpenoids from this plant. As a result, a total of five new isopimarane and 3,4-*seco* isopimarane diterpenes [isoamethinols A–E (**1**–**5**)], along with the known compound 3,4-*seco* isopimara-4(18),7,15-triene-3-oic acid methylester (**6**) [11], were isolated from the leaves of *I. amethystoides* (Fig. 1). The newly isolated compounds were subsequently tested for their cytotoxic, antibacterial, and antiviral activities. Herein, we describe the isolation, structural determination, and biological activity evaluation of the compounds obtained from *I. amethystoides*.

## Result and discussion

**Fig. 2:**
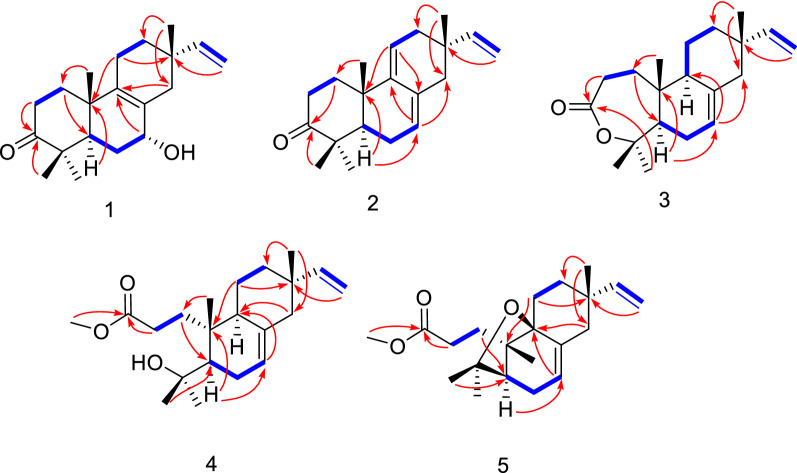
The selected key ^1^H-^1^H COSY (blue bold) and HMBC (red arrows) correlations of **1**–**5**

**Fig. 3:**
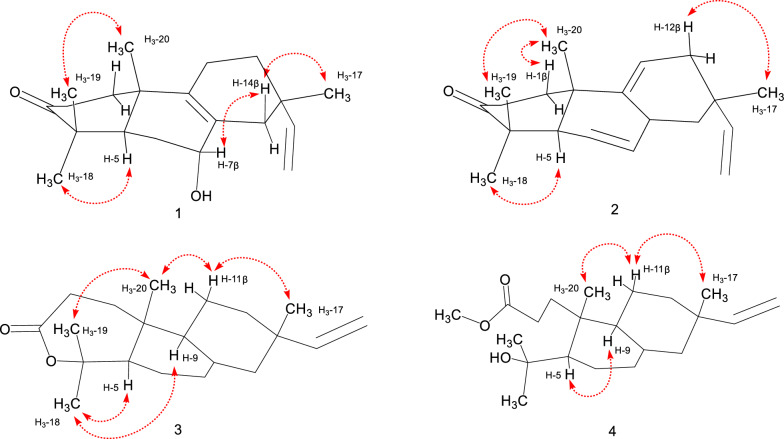
The key NOESY correlations (red arrows) of **1**–**4**

**Fig. 4:**
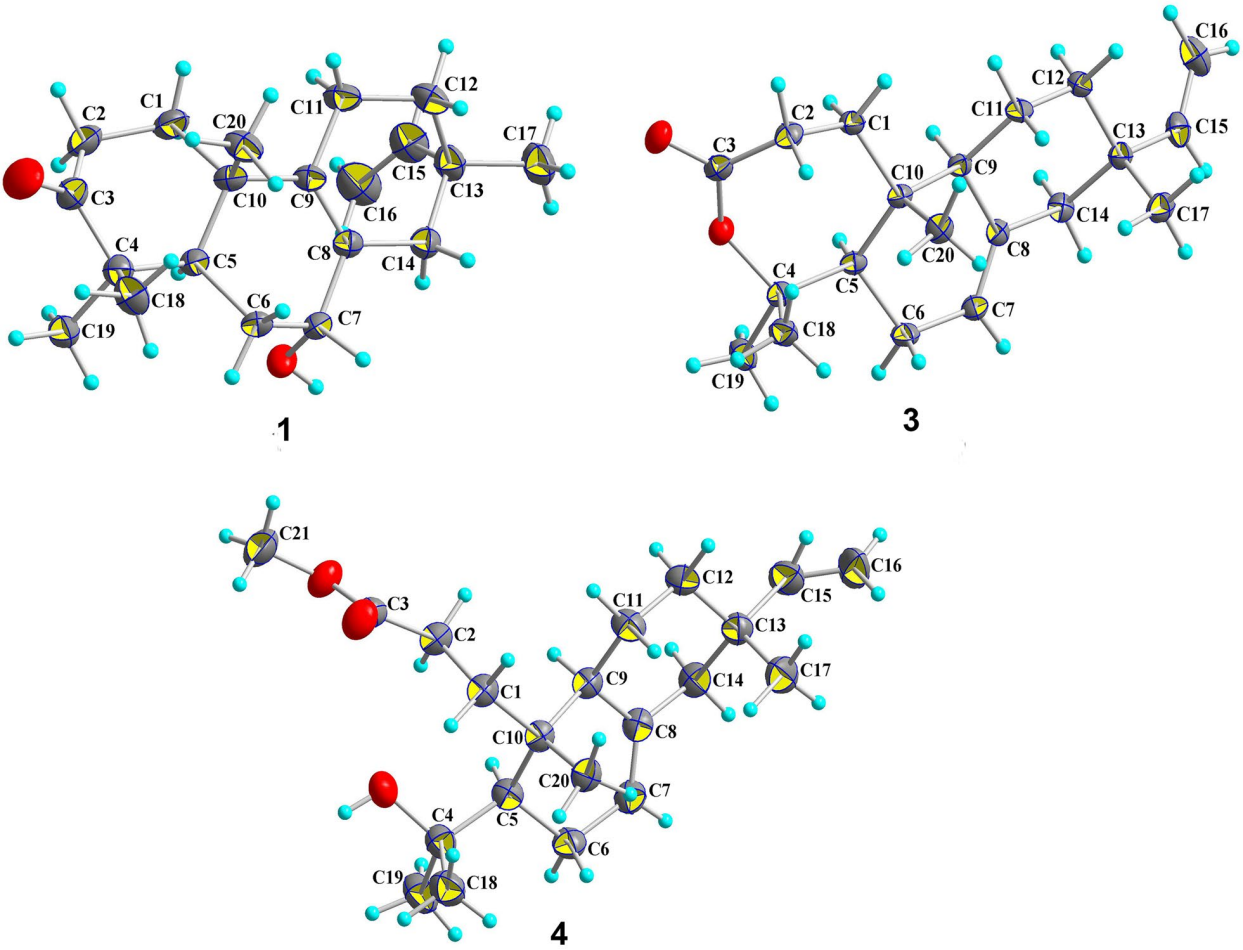
X-ray crystallographic structures of **1**, **3** and **4**

Compound **1** was obtained as a colorless cubic crystal (MeOH). It has 6 degrees of unsaturation according to the deduced molecular formula of C_20_H_30_O_2_ by analysis of the HR-ESI-MS data [*m*/*z* 325.2133 [M + Na]^+^ (calcd for 325.2138)]. By interpreting the chemical shift signals in the high and middle field regions of the ^1^H-NMR spectrum (Table 1), **1** was found to possess four methyl groups (3H, *δ*_H_ 1.00, s; 1.01, s; 1.06, s; 1.12, s) and three olefinic groups (1H, *δ*_H_ 5.72, dd, *J* = 17.5, 10.8; 4.96, dd, *J* = 17.5, 1.3; 4.93, dd, *J* = 10.8, 1.3). Moreover, analyses of the 1D ^13^C and DEPT-135 NMR data (Table 2), combined with the HSQC correlation data, allowed to identify the 20 carbon resonances in **1** as one carbonyl carbon (*δ*_C_ 217.5), five quaternary carbons including two non-protonated olefinic carbons (*δ*_C_ 127.5 and 140.0), one methine (*δ*_C_ 45.3), one oxygenated methine (*δ*_C_ 68.8), six methylenes (*δ*_C_, 34.7; 34.4; 29.5; 21.3; 34.6; 39.2), two protonated olefinic carbons (*δ*_C_ 145.8 and 111.6), and four methyl groups (*δ*_C_ 28.1; 26.7; 21.3; 17.9). The above NMR spectral data suggested that compound **1** belongs to a pimarane or an isopimarane diterpenoid. Based on the analysis of the correlations obtained from the ^1^H-^1^H COSY and HMBC spectra (Fig. 2), the ketone carbon at *δ*_C_ 217.5 is assigned at C-3, the four olefinic carbons at *δ*_C_ 127.5/140.0 and *δ*_C_ 111.6/145.8 are assigned as the two double bonds of Δ^8,9^ and Δ^15,16^ respectively, and the oxygenated methine carbon at *δ*_C_ 68.8 is assigned at C-7. The relative configuration of **1** was established through the analysis of the NOE correlations obtained from the NOESY spectrum of **1**. First of all, the presence of the NOE correlations between H-5 and H_3_-18 and between H_3_-19 and H_3_-20 indicated the possibility of a *trans*-fused A/B ring system (Fig. 3). In addition, the observed NOSEY cross-peaks between H-7 and H-14*β* and between H-14*β* and H_3_-17 determined the co-facial relationship of the protons of H-7 and H_3_-17. The resulted Flack number of 0.07 (10) from the refinement of the X-ray crystallographic data on Cu*Kα* radiation allows the determination of the absolute configuration of **1** to be the same as an isopimarane diterpenoid (Fig. 4). The structure of compound **1** was thus assigned as isopimara-7*α*-hydroxy-8,15-diene-3-one, and given the trivial name isoamethinol A.

**Table 1:** ^1^H NMR data for **1**–**5** (*δ* in ppm)

Position	*δ*_H_, mult, (*J* in Hz)				
	**1**	**2**	**3**	**4**	**5**
1*α*	1.60, dt (13.2, 8.5)	1.75, td (14.2, 4.3)	1.57, overlap		
1a				1.75, ddd (17.4, 11.2, 5.4)	2.26, dd (12.0, 4.1)
1*β*	1.98, overlap	2.17, dd (5.5, 3.2)	1.97, td (8.1, 1.9)		
1b				2.28, dt (11.3, 2.7)	2.45, ddd (15.3, 11.9, 5.7)
2*α*	2.48, ddd (16.1, 8.0, 4.1)	2.31, td (14.5, 3.7)	2.65, ddd (16.8, 8.6, 1.5)		
2a				2.22, ddd (14.7, 11.4, 5.3)	1.36, ddd (14.0, 11.9, 5.7)
2*β*	2.53, ddd (16.1, 9.8, 7.6)	2.78, dt (14.7, 5.4)	2.73, ddd (16.7, 11.3, 1.7)		
2b				2.70, ddd (14.3, 11.0, 2.0)	2.01, overlap
5*α*	1.95, overlap	1.59, dd (11.5, 4.4)	1.80, dd (11.8, 4.1)	1.65, dd (11.8, 4.5)	1.77, s
6*α*	1.78, m	2.07, t (5.0)	2.03, dtt (16.5, 6.0, 2.1)	1.98, br d (13.7)	2.05, dt (15.3, 2.9)
6*β*	1.73 dt (13.9, 2.5)	2.22, td (5.4, 3.1)	1.94, td (8.5, 3.1)	1.94, dd (13.6, 2.5)	2.20, overlap
7*β*	3.94, s	5.47, d (5.2)	5.38, dt (3.9, 1.6)	5.30, dd (5.3, 1.9)	5.37, dd (4.7, 2.3)
9*α*	–	–	1.79, d (3.9)	1.89, overlap	–
11*α*	1.95, overlap	5.41, t (4.0)	1.36, dq (13.7, 3.3)	1.40, dd (13.4, 3.4)	1.56, overlap
11*β*	1.95, overlap		1.61, overlap	1.54, ddd (12.6, 6.5, 2.8)	1.56, overlap
12*α*	1.37, ddd (12.8. 9.8, 5.9)	2.13, overlap	1.37, dt (14.3, 12.5)	1.35, d (7.4)	1.45, dt (5.7, 2.7)
12*β*	1.55, dddd (12.8, 5.7, 4.2, 1.7)	1.99, dd (17.9, 3.5)	1.51, dt (9.4, 2.9)	1.49, dt (9.2, 2.7)	1.52, overlap
14*α*	1.76, dd (12.6, 4.1)	2.13, overlap	1.94, d (2.5)	1.98, brd (13.7)	2.23, overlap
14*β*	2.39, d (17.1)	2.13, overlap	1.99, overlap	1.94, overlap	2.23, overlap
15	5.72, dd (17.5, 10.8)	5.78, dd (17.5, 10.7)	5.80, dd (17.5, 10.7)	5.80, dd (17.5, 10.7)	5.60, dd (17.6, 10.9)
16a	4.96, dd (17.5, 1.3)	4.92, dd (17.5, 1.4)	4.92, dd (14.1, 1.2)	4.91, dd (17.5, 1.2)	4.98, dd (17.6, 1.4)
16b	4.93, dd (10.8, 1.3)	4.88, dd (10.8, 1.4)	4.90, dd (11.9, 1.2)	4.87, dd (10.7, 1.2)	4.92, dd (10.9, 1.4)
17	1.00, s	0.98, s	0.87, s	0.86, s	0.96, s
18	1.12, s	1.06, s	1.42, s	1.21, s	1.28, s
19	1.06, s	1.12, s	1.55, s	1.34, s	1.27, s
20	1.01, s	1.19, s	1.03, s	1.0, s	1.13, s
OCH_3_				3.65, s	3.66, s

The ^1^H NMR (600 MHz) spectral data of **1** and **3**–**5** were measured in CDCl_3_, and the ^1^H NMR (400 MHz) spectral data of 2 were measured in CDCl_3_

**Table 2:** ^13^C NMR data for compounds **1**–**5** (*δ* in ppm)

Position	*δ*_C_, type									
	**1**		**2**		**3**		**4**		**5**	
1	34.7	CH_2_	36.5	CH_2_	33.3	CH_2_	32.3	CH_2_	31.7	CH_2_
2	34.4	CH_2_	34.9	CH_2_	31.9	CH_2_	29.3	CH_2_	30.9	CH_2_
3	217.5	C	216.7	C	175.8	C	175.7	C	174.7	C
4	46.8	C	47.8	C	86.4	C	75.1	C	81.6	C
5	45.3	CH	50.2	CH	51.7	CH	49.5	CH	49.7	CH
6	29.5	CH_2_	24.3	CH_2_	27.0	CH_2_	27.3	CH_2_	28.3	CH_2_
7	68.8	CH	122.6	CH	121.1	CH	121.4	CH	123.3	CH
8	127.5	C	131.6	C	135.8	C	135.9	C	137.9	C
9	140.0	C	145.2	C	49.4	CH	44.5	CH	81.8	C
10	37.9	C	36.7	C	38.5	C	38.9	C	46.5	C
11	21.3	CH_2_	117.1	CH	20.4	CH_2_	19.5	CH_2_	25.3	CH_2_
12	34.6	CH_2_	38.3	CH_2_	36.2	CH_2_	36.2	CH_2_	34.5	CH_2_
13	35.1	C	36.1	C	36.9	C	36.9	C	36.5	C
14	39.2	CH_2_	43.5	CH_2_	46.2	CH_2_	46.1	CH_2_	41.7	CH_2_
15	145.8	CH	146.4	CH	150.0	CH	150.3	CH	145.4	CH
16	111.6	CH_2_	110.9	CH_2_	109.1	CH_2_	109.5	CH_2_	113.4	CH_2_
17	28.1	CH_3_	26.5	CH_3_	21.6	CH_3_	21.6	CH_3_	30.0	CH_3_
18	26.7	CH_3_	24.5	CH_3_	34.1	CH_3_	34.2	CH_3_	31.2	CH_3_
19	21.3	CH_3_	22.4	CH_3_	24.3	CH_3_	27.0	CH_3_	27.4	CH_3_
20	17.9	CH_3_	20.8	CH_3_	15.4	CH_3_	18.2	CH_3_	22.6	CH_3_
OCH_3_							51.7	CH_3_	51.7	CH_3_

The ^13^C NMR (150 MHz) spectral data of **1** and **3**–**5** were measured in CDCl_3_, and the ^13^C NMR (100 MHz) spectral data of 2 were measured in CDCl_3_

Compound **2**, colorless oil, was found to possess a molecular formula of C_20_H_28_O by the HR-ESI-MS data [*m/z* 285.2206 [M + H]^+^ (calcd for 285.2213)]. The similar coupling pattern and chemical shifts of the ^1^H and ^13^C NMR spectroscopic data to **1** were observed for **2** (Tables 1 and 2). Compound **2** differs from **1** by lacking the hydroxyl group at C-7 but having three olefinic double bonds in the structure. The three double bonds in **2** are assigned as Δ^7,8^, Δ^9,11^ and Δ^15,16^, respectively, by analyzing the 2D correlation data of the ^1^H-^1^H COSY, HMQC and HMBC NMR spectra (Fig. 2). Compound **2** shared the similar key NOE correlation pattern (H-1/H-20, H-18/H-5, H-19/H-20, H-12*β*/H-17) to **1**, revealing that **2** is also an isopimarane diterpenoid. The structure of **2** was thus established as isopimara-7,9(11),15-triene-3-one, and given the trivial name isoamethinol B.

Compound **3** was obtained as colorless crystals (MeOH). Given the HR-ESI–MS molecular ion peak at *m/z* 325.2129 [M + Na]^+^ (calcd 325.2138) and the molecular formula of C_20_H_30_O_2_, **3** is calculated to have one less degree of unsaturation than **2**. By interpretation of the correlation data of the HMBC and ^1^H-^1^H COSY spectra (Fig. 2), **3** was elucidated to be a ring-expanded ω lactone congener of **2**. Specifically, the absence of the HMBC correlations of H_3_-18 to C-3 and H_3_-19 to C-3 in **3** (Additional file 1: Figs. S25 and S26 combined with the observation of the upfield chemical shift of C-3 (δ_C_ 175.8) and a downfield chemical shift of C-4 (δ_C_ 86.4) in **3** in comparison of **2**, revealed that the ω lactone is formed via an oxygen insertion at the position between C-3 carbonyl carbon and C-4. Compared with **2**, compound **3** contains a less olefinic double bond, which is determined to occur at C-8 and C-9. These structure features in **3** are evidenced by the disappearance of the characteristic double bond Δ^9,11^ (*δ*_C_ 117.1, 145.2) resonance signals (Table 2), the upfield shift of the carbonyl carbon (from *δ*_C_ 216.6 to 175.8) and the downfield shift of the gem methyl group connected quaternary carbon (from *δ*_C_ 47.8 to 86.4) in comparison with **2**. It is worthy to mention that a pimarane diterpene with ring A being oxidated to a lactone like **3** is rarely discovered in nature. The relative stereochemistry of **3** was determined by a NOESY experiment. The presence of the NOE cross-peaks of H_3_-20/H-11*α*, H_3_-20/H_3_-19 and H-11*α*/H_3_-17 demonstrated that the three methyl groups of C-17, C-19 and C-20 are co-facial and *β*-oriented. H-5 and H-9 were established as *α*-oriented by the observation of the NOE correlations between H-5 and H_3_-18 and between H-9 and H_3_-18. In addition, the X-ray diffraction results further confirmed the structure elucidated from the above deduction (Fig. 4). As a result, **3** was identified as isopimara-7,15-diene-3,4-lactone and given the trivial name isoamethinol C.

Compound **4**, colorless needle crystals (MeOH), was determined to possess 5 degrees of unsaturation calculated by the molecular formula of C_21_H_34_O_3_, which was obtained through the analysis of its HR-ESI-MS data [*m/z* 357.2397 [M + Na]^+^ (calcd for C_21_H_34_O_3_Na, 357.2400)]. It has one unsaturation degree less than **3**. The complete assignments of the 1D and 2 D NMR signals (Tables 1 and 2, and Fig. 2) allowed us to identify a methyl propanoate ester group [*δ*_H_ 3.65 (3H, s, OCH_3_), 1.75 (1H, ddd, *J* = 17.4, 11.2, 5.4, H-1*α*), 2.28 (1H, dt, *J* = 11.3, 2.7, H-1*β*), 2.22 (1H, ddd, *J* = 14.0, 11.9, 5.7, H-2*α*), 2.70 (1H, ddd, *J* = 14.3, 11.0, 2.0, H-2*β*); *δ*_C_ 51.7 (OCH_3_), 32.3 (C-1), 29.3 (C-2), 175.7 (C-3, the ester carbonyl carbon)] linked to C-10. The analysis of the NMR data also assigned an oxygenated tertiary carbon at C-4, which is attached with two gem methyl groups to form a 2-hydroxy propanyl group. Collectively, compound **4** was elucidated as a ring A opened methyl ester derivative. The key NOE correlations observed between H-5 and H-9 and between H-20 and H-11*β* determined that the propanoate ester group and the 2-hydroxy propanyl group are *trans*-oriented with each other. Moreover, the presence of the cross-peaks of H_3_-17/H-11*β* and H_3_-20/H-11*β* revealed the *β*-orientation of the methyl groups at C-10 and C-13. Finally, a single-crystal X-ray diffraction study (Cu*Kα* radiation) with a Flack parameter of − 0.01(6) determined the absolute configuration of **4** shown in Fig. 4. Accordingly, **4** was determined as 3,4-*seco*-isopimara-4-hydroxy-7,15-diene-3-oic acid methylester, and given the trivial name isoamethinol D.

Compound **5** was obtained as yellow oil. The analysis of the molecular ion peak [M + H]^+^ at *m/z* 333.2420 (calcd 333.2424) measured in the HR-ESI-MS spectrum rendered the molecular formula of **5** as C_21_H_32_O_3_, which has 6 degrees of unsaturation and two protons less than that of **4**. Compound **5** showed very similar ^1^H and ^13^C NMR spectral data to 4. The one more degree of unsaturation of **5** than **4** suggested that additional ring could be formed in **5**. Through the total analysis of the 1D and 2D NMR data (Tables 1 and 2, and Fig. 2), the additional ring was found to be formed by the bond connection between C-4 and C-9. Moreover, **5** shared high similarity of the proton NMR spectral data to our previously identified compound fladin A, except for an additional methoxy group (3H, *δ*_H_ 3.66, s, *δ*_C_ 51.7) observed in **5** [17]. Herein, compound **5** is a methyl ester congener of fladin A. The absolute configuration of **5** was assigned the same as **4**, which was deduced by the consideration of the biosynthetic origins and the similar NOE correlation patterns observed between the two compounds. Compound **5** was thus identified as 3,4-*seco* isopimara-4,9*β*-epoxy-7,15-diene-3-oic acid methylester, and given the trivial name isoamethinol E.

Compound **6** was identified as the known 3,4-*seco* isopimara-4(18),7,15-triene-3-oic acid methylester based on the comparison of the proton coupling patterns and chemical shifts of the ^1^H and ^13^C NMR data (Additional file 1: Figs. S50 and S51) with the reported literature data [11].

**Fig. 5:**
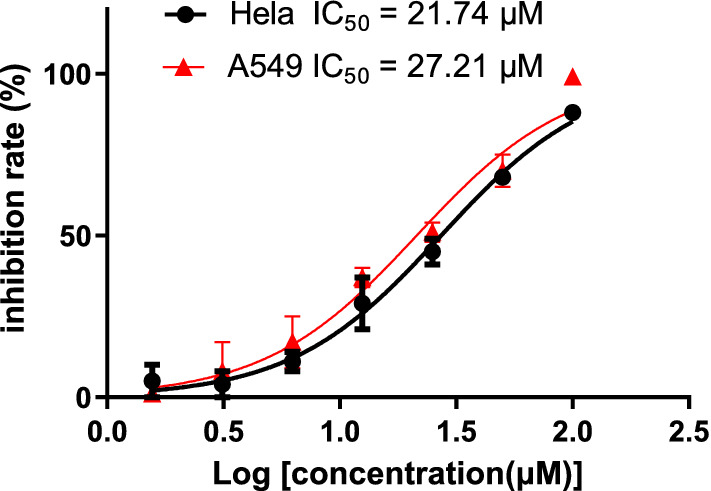
Growth inhibitory effects of compound **4** on Hela and A549 cancer cells. The in vitro dose–response was evaluated in the cells at seven twofold serial dilution concentrations with 3 independent experiments. The IC_50_ values were determined by fitting dose–response curves with the equation log (inhibitor) vs. normalized response–variable slope to the data by using GraphPad Prism software

Compounds **1**–**5** were evaluated for their in vitro cytotoxic activities against a panel of human cancer cell lines (HL-60 promyelocytic leukemia cells, SMMC-7721 hepatocellular cancer cells, MCF-7 breast cancer cells, SW480 colon cancer cells, and Hela cervical carcinoma cells). Among these compounds, only **4** showed moderate cytotoxicities against Hela and A549 cells with IC_50_ values of 27.21 and 21,47 μM, respectively (Fig. 5). These compounds were also evaluated for their activities against the gram-positive bacteria including methicillin-resistant strain *Staphylococcus aureus* (MRSA), *Streptococcus sobrinus,* and *S. mutans*. As a result, only **4** was demonstrated with mild antibacterial effect on *S. mutans* with an inhibitory rate of 42.1% at the concentration of 149.48 μM. In addition, the anti-HIV activities of these compounds were further explored for their antiviral potential using our previously established “One-Stone-Two-Birds” protocol [12]. However, no significant antiviral activities were observed for these compounds at a concentration of 25 μM.

## Conclusion

Five new isopimarane and 3,4-*seco* isopimarane diterpenes, along with a known 3,4-*seco* isopimarane diterpene, were isolated from the leaves of *I. amethystoides*. Their chemical structures and absolute configurations were determined by analysing their spectroscopic and X-ray crystallographic data. To the best of our knowledge, isoamethinol C (**3**) is the first naturally occurring 3,4-*seco* isopimarane diterpenoid containing a A-ring ω lactone ring, which expands the chemical diversity of isopimarane diterpenoid family. Isoamethinol D (**4**) showed moderate cytotoxicity and mild antibacterial activity. The present findings through the phytochemical investigation including the absolute structure confirmation and the bioactivity evaluation of the new isolates suggested that the structurally diversified isopimarane diterpenes derived from *I. amethystoides* could serve as a class of lead molecules for further drug discovery study.

## Experimental section

### General

Optical rotation was measured at 28.6 °C for compounds **1**–**5** with a WZZ-3 automatic polarimeter (INESA Analytical Instrument, Shanghai, People’s Republic of China). An ICAN-9 Fourier transform infrared spectrometer was used for scanning IR spectroscopy using KBr discs. Nuclear magnetic resonance (NMR) experiments were carried out on a Bruker Advance NEO (600 MHz, Brucker, Karlsruhe, Germany) spectrometer and an INOVA (400 MHz, Varian, Palo Alto, USA) spectrometer using TMS as an internal standard. X-ray crystallographic data were obtained on a Bruker SMART *APEX* CCD instrument using Mo*Kα* radiation for compound **3** and on a Bruker D8 VENTURE instrument using Cu*Kα* radiation for compound **1** and **4** (Brucker, Karlsruhe, Germany). HR-ESI-MS data were recorded on a Bruker Daltonics Compact Q-TOF spectrometer (Brucker, Karlsruhe, Germany) equipped with an ACQUITY UPLC BEH C18 column (2.1 × 50 mm, 1.7 µm; Waters, Milford, MA, USA) under the column heater fixed at 40 °C. Silica gel (100–200 and 200–300 mesh, Qingdao Marine Chemical Inc., Qingdao, People’s Republic of China) was used for flash chromatography. Sephadex LH-20 gel (Shanghai EKEAR Bio@Tech, Shanghai, People’s Republic of China) was used for chromatographic separation. Thin-layer chromatography (TLC) analyses were carried out on precoated silica gel GF_254_ plates (Qingdao Marine Chemical Inc., Qingdao, People’s Republic of China) using various solvent system, and spots were visualized by heating the silica gel plates sprayed with 95–98% H_2_SO_4_-EtOH (v/v = 10:90). All solvents were commercially purchased and distilled prior to use.

### Plant material

The leaves of *I. amethystoides* were collected in October 2018 from Libo County in Guizhou Province of China. The collected plant was authenticated by Professor Junhua Zhao from Guizhou University of Traditional Chinese Medicine. A voucher plant specimen (GZLB20181003004) was deposited at Guizhou University of Traditional Chinese Medicine.

### Extraction and isolation

The air-dried leaves of *I. amethystoides* (29 kg) were ground into powder and further extracted by MeOH at room temperature (3 times, 7 days each time) to yield a crude extract (3.2 kg), which was suspended in water (8 L), and partitioned with petroleum ether (PE) (3 × 8 L, 48 h each time) and ethyl acetate (EtOAc) (3 × 8 L, 48 h each time). The PE fraction (1.5 kg) was subjected to a silica gel column separation with gradient elution of PE/EtOAc (1:0 to 1:1) to provide fractions A–D. Fraction B (234 g) was further separated on a silica gel column using a gradient elution of PE/dichloromethane (CH_2_Cl_2_) (99:1 to 1:1) to afford compound **6** (160 mg). Fraction C (345 g) was separated on a silica gel column, eluting with gradient PE/EtOAc (49:1 to 1:1) to obtain **2** (18 mg) and **5** (200 mg). The EtOAc fraction (880 g) was subjected to fractionation over an MCI gel column using gradient MeOH/H_2_O (50:50 to 90:10) to yield fractions E–I. Fraction G (179 g) was subjected to a silica gel chromatographic separation using gradient (EtOAc/MeOH) (50:1 to 5:1) to produce sub-fractions G1–G4. G1 was subjected to a Sephadex LH-20 chromatographic column eluting with CH_2_Cl_2_/MeOH (1:1) to give **1** (23 mg). Fraction H (191 g) was further purified by a silica gel column, eluting with gradient CH_2_Cl_2_/MeOH (50:1 to 1:1) to afford **3** (156 mg) and **4** (90 mg).

#### Isoamethinol A (1)

Colorless cubic crystal, mp 88.6–89.1 °C; [*α*]^28.6^_D_ + 46.67 (*c* 0.6, CH_2_Cl_2_); IR (KBr) *υ*_max_ 3522, 2935, 2921, 2873, 1690, 1458, 1454, 1383, 1245, 1040, 932, 909 cm^–1^; ^1^H and ^13^C NMR (CDCl_3_), see Tables 1 and 2; HR-ESI-MS *m/z* 325.2133 [M + Na]^+^ (calcd. for C_20_H_30_O_2_Na, 325.2138).

#### Isoamethinol B (2)

Colorless oil; [*α*]^28.6^_D_ + 69.54 (*c* 0.3, CH_2_Cl_2_); IR (KBr) *υ*_max_ 3404, 2964, 1709, 1458, 1386, 1271, 1116, 1006, 910, 803 cm^–1^; ^1^H and ^13^C NMR (CDCl_3_), see Tables 1 and 2; HR-ESI-MS *m/z* 285.2218 [M + H]^+^ (calcd. for C_20_H_29_O, 285.2218).

#### Isoamethinol C (3)

Colorless needle crystal, mp 83.3–83.6 °C; [*α*]^28.6^_D_-15.38 (*c* 0.26, CH_2_Cl_2_); IR (KBr) *υ*_max_ 3421, 2957, 2918, 1726, 1639, 1389, 1371, 1225, 1108, 986, 910 cm^–1^; ^1^H and ^13^C NMR (CDCl_3_), see Tables 1 and 2; HR-ESI-MS *m/z* 325.2129 [M + Na]^+^ (calcd. for C_20_H_30_O_2_Na, 325.2138).

#### Isoamethinol D (4)

Colorless needle crystal, mp 84.36–85.3 °C; [*α*]^28.6^_D_ + 17.39 (*c* 0.23, CH_2_Cl_2_); IR (KBr) *υ*_max_ 3491, 3373, 2905, 2842, 1732, 1638, 1439, 1411, 1251, 1198, 1001, 912 cm^–1^; ^1^H and ^13^C NMR (CDCl_3_), see Tables 1 and 2; HR-ESI-MS *m/z* 357.2397 [M + Na]^+^ (calcd. for C_21_H_34_O_3_Na, 357.2400).

#### Isoamethinol E (5)

Yellow oil; [*α*]^28.6^_D_ + 38.10 (*c* 0.53, CH_2_Cl_2_); IR (KBr) *υ*_max_ 2359, 2342, 1741, 1173, 913 cm^–1^; ^1^H and ^13^C NMR (CDCl_3_), see Tables 1 and 2; HR-ESI-MS *m/z* 357.2397 [M + Na]^+^ (calcd. for C_21_H_34_O_3_Na, 357.2400).

### X-ray crystallographic data of 1, 3 and 4

Crystals of **1**, **3**, and **4** were obtained from MeOH. Single-crystal X-ray crystallographic analyses of **1** and **4** were obtained on a Bruker D8 VENTURE diffractometer (Cu *Kα* radiation, *λ* = 1.54178 Å). The crystal structure of 3 was determined on a Bruker APEX-II CCD diffractometer (Mo *Kα* radiation, *λ* = 0.71076 Å). The crystals of **1**, **3**, and **4** were kept at 160.0 K, 293.0 (2) K, and 170.0 K during data collection, respectively. Using Olex2, the structures were solved with the ShelXT structure solution program using direct methods, dual space, or intrinsic phasing, and refined with the ShelXL refinement package using least-squares minimization [13–15].

#### Crystallographic data of 1

C_20_H_30_O_2_, *M* = 302.44, *a* = 9.1730 (2) Å, *b* = 11.5454 (3) Å, *c* = 16.8349 (4) Å; *α* = 90^◦^, *β* = 90^◦^, *γ* = 90^◦^, *V* = 1782.92 (6) Å^3^, *T* = 160.0 K, space group *P*2_1_2_1_2_1_, *Z* = 4, *μ* (Cu *Kα*) = 0.54 mm^−1^; 49814 reflections collected, 3644 independent reflections (R_*int*_ = 0.0617, R_*sigma*_ = 0.0275). The final *R*_*1*_ values were 0.0438 [*I* > 2*σ* (*I*)]. The final *wR* (*F*^*2*^) values were 0.1161 [*I* > 2*σ* (*I*)]. The final *R*_*1*_ values were 0.0484 (all data). The final wR (*F*^*2*^) values were 0.1188 (all data). The goodness of fit on *F*^*2*^ was 1.049. Flack parameter = 0.07 (10).

#### Crystallographic data of 3

C_20_H_30_O_2_, *M* = 302.44, *a* = 6.238 (2) Å, *b* = 9.613 (4) Å, *c* = 29.043 (13) Å; *α* = 90^◦^, *β* = 90^◦^, *γ* = 90^◦^, *V* = 1782.92 (6) Å^3^, *T* = 293.0 (2) K, space group *P*2_1_2_1_2_1_, *Z* = 4, *μ* (Mo K*α*) = 0.07 mm^−1^; 18262 reflections collected, 3072 independent reflections (R_*int*_ = 0.0203, R_*sigma*_ = 0.0133). The final *R*_*1*_ values were 0.0290 [*I* > 2*σ* (*I*)]. The final *wR* (*F*^*2*^) values were 0.0720 [*I* > 2*σ* (*I*)]. The final *R*_*1*_ values were 0.0297 (all data). The final wR (*F*^*2*^) values were 0.0725 (all data). The goodness of fit on *F*^*2*^ was 1.055. Flack parameter = − 0.2 (3).

#### Crystallographic data of 4

C_21_H_34_O_3_, *M* = 334.48, *a* = 13.8691 (10) Å, *b* = 10.3267 (7) Å, *c* = 14.8423 (11) Å; *α* = 90^◦^, *β* = 107.992(3), *γ* = 90^◦^, *V* = 2021.8 (3) Å^3^, *T* = 170.0 K, space group *P*2_1_, *Z* = 4, *μ* (Cu *Kα*) = 0.56 mm^−1^; 44189 reflections collected, 8085 independent reflections (R_*int*_ = 0.0531, R_*sigma*_ = 0.0323). The final *R*_*1*_ values were 0.0381 [*I* > 2*σ* (*I*)]. The final *wR* (*F*^*2*^) values were 0.1042 [*I* > 2*σ* (*I*)]. The final *R*_*1*_ values were 0.0443 (all data). The final wR (*F*^*2*^) values were 0.1086 (all data). The goodness of fit on *F*^*2*^ was 1.048. Flack parameter = − 0.01 (6).

### Cytotoxicity assay

A panel of human cancer cell lines (HL-60, SMMC-7721, MCF-7, SW480, and Hela) were utilized to determine the cytotoxic effects of compounds **1**–**5** using MTS method as previously reported [16]. HL-60, MCF-7, and Hela cells were cultured in DMEM medium supplemented with 10% fetal bovine serum (FBS), where the SMMC-7721 and SW480 cells were cultured in RPMI-1640. Cells were seeded at 4000 cells/190 µL per well in 96-well plates in their corresponding medium before adding 10 µL test samples. Serial concentrations (1.25–40 µM) of each tested compound were added in wells, and the plates were incubated for 48 h. Spent media was decanted after 48 h incubation. To each well, 100 µL of fresh media and 20 µL of 2 mg/mL MTS stock solution were added and the cells were incubated for 3 h at 37 °C. Afterwards, the optical density was measured at 515 nm using an ELISA plate reader. Both cisplatin and paclitaxel were used as positive controls.

### Antibacterial assay

The bacteriostatic activity assay for compounds **1**–**5** was carried out using the standard microdilution method in 96-well plates as previously described [17]. Each well contained 50 μL two-fold serially diluted test agents, 50 μL growth medium (Tryptic Soy Broth Medium for MRSA, Brain Heart Infusion for *S. mutans* and *S. sobrinus*, respectively), and 10 μL of an overnight culture of strains, representing approximately 5 × 10^7^ CFU mL^−1^. The controls comprised inoculated growth medium without test agents and sample blanks in growth medium only. The plates were incubated overnight at 200 rpm, 37 °C. The OD_600_ values were obtained using the microplate reader.

### Anti-HIV assay

Compounds **1** to **5** were also evaluated for their anti-HIV activity using our previously established “One-Stone-Two-Birds” protocol [18]. Human embryonic kidney cells 293 T were transiently transfected with 0.5 μg VSV-G envelope expression plasmid and 2 μg Env-deficient HIV vector (pNL4.3. Luc-R-E-) in 10 cm plates via PEI (polyethyleneimine). Sixteen hours post-transfection, all media was replaced with fresh, complete DMEM. Forty-eight hours post-transfection, the supernatants were collected and filtered through a 0.45 μm-pore size filter, and the pseudovirions were directly used for infection. Targeted A549 cells were seeded at 4000 cells/190 µL per well in 96-well plate in DMEM supplemented with 10% FBS. The tested samples were added, 10 μL in each well, with a final concentration of 5 and 25 μM. Forty-eight hours post-infection, cells were lysed and prepared for luciferase assay.

## Supplementary Information

**Additional file 1: Figure S1.** ^1^H NMR (600 MHz, CDCl_3_) spectrum of isoamethinol A (1). **Figure S2.** ^13^C NMR (150 MHz, CDCl_3_) spectrum of isoamethinol A (1). **Figure S3.** DEPT spectrum of isoamethinol A (1). **Figure S4.** HSQC spectrum of isoamethinol A (1). **Figure S5.** HMBC spectrum of isoamethinol A (1). **Figure S6.** ^1^H-^1^H COSY spectrum of isoamethinol A (1). **Figure S7.** NOESY spectrum of isoamethinol A (1). **Figure S8.** IR spectrum of isoamethinol A (1). **Figure S9.** HR-ESI-MS spectrum of isoamethinol A (1). **Figure S10.** UV spectrum of isoamethinol A (1). **Figure S11.** ^1^H NMR (600 MHz, CDCl_3_) spectrum of isoamethinol B (2). **Figure S12.** ^13^C NMR (150 MHz, CDCl_3_) spectrum of isoamethinol B (2). **Figure S13.** DEPT spectrum of isoamethinol B (2). **Figure S14.** HSQC spectrum of isoamethinol B (2). **Figure S15.** HMBC spectrum of isoamethinol B (2). **Figure S16.** ^1^H-^1^H COSY spectrum of isoamethinol B (2). **Figure S17.** NOESY spectrum of isoamethinol B (2). **Figure S18.** IR spectrum of isoamethinol B (2). **Figure S19.** HR-ESI-MS spectrum of isoamethinol B (2). **Figure S20.** UV spectrum of isoamethinol B (2). **Figure S21.** ^1^H NMR (600 MHz, CDCl_3_) spectrum of isoamethinol C (3). **Figure S22.** ^13^C NMR (150 MHz, CDCl_3_) spectrum of isoamethinol C (3). **Figure S23.** DEPT spectrum of isoamethinol C (3). **Figure S24.** HSQC spectrum of isoamethinol C (3). **Figure S25.** HMBC spectrum of isoamethinol C (3). **Figure S26.** ^1^H-^1^H COSY spectrum of isoamethinol C (3). **Figure S27.** NOESY spectrum of isoamethinol C (3). **Figure S28.** IR spectrum of isoamethinol C (3). **Figure S29.** HR-ESI-MS spectrum of isoamethinol C (3). **Figure S30.** ^1^H NMR (600 MHz, CDCl_3_) spectrum of isoamethinol D (4). **Figure S31.** ^13^C NMR (150 MHz, CDCl_3_) spectrum of isoamethinol D (4). **Figure S32.** DEPT spectrum of isoamethinol D (4). **Figure S33.** HSQC spectrum of isoamethinol D (4). **Figure S34.** HMBC spectrum of isoamethinol D (4). **Figure S35.** ^1^H-^1^H COSY spectrum of isoamethinol D (4). **Figure S36.** NOESY spectrum of isoamethinol D (4). **Figure S37.** IR spectrum of isoamethinol D (4). **Figure S38.** HR-ESI-MS spectrum of isoamethinol D (4). **Figure S39.** UV spectrum of isoamethinol D (4). **Figure S40.** ^1^H NMR (600 MHz, CDCl_3_) spectrum of isoamethinol E (5). **Figure S41.** ^13^C NMR (150 MHz, CDCl_3_) spectrum of isoamethinol E (5). **Figure S42.** DEPT spectrum of isoamethinol E (5). **Figure S43.** HSQC spectrum of isoamethinol E (5). **Figure S44.** HMBC spectrum of isoamethinol E (5). **Figure S45.** ^1^H-^1^H COSY spectrum of isoamethinol E (5). **Figure S46.** NOESY spectrum of isoamethinol E (5). **Figure S47.** IR spectrum of isoamethinol E (5). **Figure S48.** HR-ESI-MS spectrum of isoamethinol E (5). **Figure S49.** UV spectrum of isoamethinol E (5). **Figure S50.** ^1^H NMR spectrum of compound **6**. **Figure S51.** ^13^C NMR spectrum of compound **6**.

## Data Availability

All data generated or analysed during this study are included in this published article and its Additional file 1.

## Abbreviations

ROR*γ*t: Related orphan receptor gamma t
MeOH: Methanol
HR-ESI-MS: High-resolution electrospray ionisation mass spectrometry
calcd: Calculated
NMR: Nuclear magnetic resonance
DEPT: Distortion less enhancement by polarization transfer
HSQC: Heteronuclear single quantum coherence spectroscopy
HMBC: Heteronuclear multiple bond coherence
COSY: Correlation spectroscopy
NOESY: Nuclear overhauser effect spectroscopy
MHz: Megahertz
EtOH: Ethanol
Q-TOF: Quadrupole time-of-flight
TLC: Thin-layer chromatography
PE: Petroleum ether
EtOAc: Ethyl acetate
CH_2_Cl_2_: Dichloromethane
CDCl_3_: Deuterated chloroform
MTS: 3-(4,5-Dimethylthiazol-2-yl)-5-(3-carboxymethoxyphenyl)-2-(4-sulfophenyl)-2H-tetrazolium
DMEM: Dulbecco’s modified eagle medium
FBS: Fetal bovine serum
ELISA: Enzyme-linked immunosorbent assay
MRSA: Methicillin-resistant *Staphylococcus aureus*
