# Supplementary material for: Bioactive isopimarane and 3,4-seco isopimarane diterpenoids from Isodon amethystoides
Source: BMC Chem. 2022 Nov 14;16(1):96. doi: 10.1186/s13065-022-00880-4 (PMC9661758; doi:10.1186/s13065-022-00880-4)
Supplement: Supplementary file 1 — Additional file 1: Figure S1. 1H NMR (600 MHz, CDCl3) spectrum of isoamethinol A (1). Figure S2. 13C NMR (150 MHz, CDCl3) spectrum of isoamethinol A (1). Figure S3. DEPT spectrum of isoamethinol A (1). Figure S4. HSQC spectrum of isoamethinol A (1). Figure S5. HMBC spectrum of isoamethinol A (1). Figure S6. 1H-1H COSY spectrum of isoamethinol A (1). Figure S7. NOESY spectrum of isoamethinol A (1). Figure S8. IR spectrum of isoamethinol A (1). Figure S9. HR-ESI-MS spectrum of isoamethinol A (1). Figure S10. UV spectrum of isoamethinol A (1). Figure S11. 1H NMR (600 MHz, CDCl3) spectrum of isoamethinol B (2). Figure S12. 13C NMR (150 MHz, CDCl3) spectrum of isoamethinol B (2). Figure S13. DEPT spectrum of isoamethinol B (2). Figure S14. HSQC spectrum of isoamethinol B (2). Figure S15. HMBC spectrum of isoamethinol B (2). Figure S16. 1H-1H COSY spectrum of isoamethinol B (2). Figure S17. NOESY spectrum of isoamethinol B (2). Figure S18. IR spectrum of isoamethinol B (2). Figure S19. HR-ESI-MS spectrum of isoamethinol B (2). Figure S20. UV spectrum of isoamethinol B (2). Figure S21. 1H NMR (600 MHz, CDCl3) spectrum of isoamethinol C (3). Figure S22. 13C NMR (150 MHz, CDCl3) spectrum of isoamethinol C (3). Figure S23. DEPT spectrum of isoamethinol C (3). Figure S24. HSQC spectrum of isoamethinol C (3). Figure S25. HMBC spectrum of isoamethinol C (3). Figure S26. 1H-1H COSY spectrum of isoamethinol C (3). Figure S27. NOESY spectrum of isoamethinol C (3). Figure S28. IR spectrum of isoamethinol C (3). Figure S29. HR-ESI-MS spectrum of isoamethinol C (3). Figure S30. 1H NMR (600 MHz, CDCl3) spectrum of isoamethinol D (4). Figure S31. 13C NMR (150 MHz, CDCl3) spectrum of isoamethinol D (4). Figure S32. DEPT spectrum of isoamethinol D (4). Figure S33. HSQC spectrum of isoamethinol D (4). Figure S34. HMBC spectrum of isoamethinol D (4). Figure S35. 1H-1H COSY spectrum of isoamethinol D (4). Figure S36. NOESY spectrum of isoamethinol D (4). Figure S37. IR spectrum of isoamethin [file 13065_2022_880_MOESM1_ESM.docx]

**Supporting information for**

**Bioactive isopimarane and 3,4-*seco* isopimarane diterpenoids from *Isodon amethystoides***

Chenliang Zhao^1,2†^, Lang Zhou^1†^, Wenjian Xie^2†^, Lihan Zhao^2^, Chiyuan Zhang^2^, Kang He^1^, Jianghai Ye^1^, Jingjie Zhang^1^, Lutai Pan^1^, Juan Zou^1*^ and Hongjie Zhang^2*^

^1^ College of Pharmacy, Guizhou University of Traditional Chinese Medicine, 4 Dongqing Road, Guiyang, Guizhou, 550025, P. R. China.

^2^School of Chinese Medicine, Hong Kong Baptist University, 7 Baptist University Road, Kowloon Tong, Kowloon, Hong Kong SAR, P. R. China.

^†^Chenliang Zhao, Lang Zhou and Wenjian Xie contributed equally to this work

**Abstract**: Five new diterpenoids categorised as isopimarane and 3,4-*seco* isopimarane scaffolds [isoamethinols A-E (1-5)], along with the known 3,4-*seco* isopimara-4(18),7,15-triene-3-oic acid methylester (**6**) were isolated from the twigs and leaves of the Chinese folk medicinal plant *Isodon amethystoides*. Their structures including absolute configurations were determined by comprehensive spectroscopic analyses and single crystal X-ray diffraction measurement. These compounds were evaluated for their biological activities against a panel of cancer cell lines, gram-positive bacterial strains and pseudotyped virus. As a result, isoamethinol D (**4**) showed moderate cytotoxic activities against human cervical cancer cells Hela with an IC_50_ of 27.21 μM and human lung cancer cells A549 with an IC_50_ of 21.47 μM, while others were measured inactive at a concentration of 20 μg/mL. Compound **4** also exhibited mild antibacterial activity against *Streptococcus mutans* (42.1% inhibitory rate at 149.48 μM).

**Keywords**: *Isodon amethystoides; folk medicine; isopimarane diterpenoids; cytotoxicity; anti-bacterial activity*

**Contents**

**Figure S1.** ^1^H NMR (600 MHz, CDCl_3_) spectrum of isoamethinol A (1).

**Figure S2.** ^13^C NMR (150 MHz, CDCl_3_) spectrum of isoamethinol A (1).

**Figure S3.** DEPT spectrum of isoamethinol A (1).

**Figure S4.** HSQC spectrum of isoamethinol A (1).

**Figure S5.** HMBC spectrum of isoamethinol A (1).

**Figure S6.** ^1^H-^1^H COSY spectrum of isoamethinol A (1).

**Figure S7.** NOESY spectrum of isoamethinol A (1).

**Figure S8.** IR spectrum of isoamethinol A (1).

**Figure S9.** HR-ESI-MS spectrum of isoamethinol A (1).

**Figure S10.** UV spectrum of isoamethinol A (1).

**Figure S11.** ^1^H NMR (600 MHz, CDCl_3_) spectrum of isoamethinol B (2).

**Figure S12.** ^13^C NMR (150 MHz, CDCl_3_) spectrum of isoamethinol B (2).

**Figure S13.** DEPT spectrum of isoamethinol B (2).

**Figure S14.** HSQC spectrum of isoamethinol B (2).

**Figure S15.** HMBC spectrum of isoamethinol B (2).

**Figure S16.** ^1^H-^1^H COSY spectrum of isoamethinol B (2).

**Figure S17.** NOESY spectrum of isoamethinol B (2).

**Figure S18.** IR spectrum of isoamethinol B (2).

**Figure S19.** HR-ESI-MS spectrum of isoamethinol B (2).

**Figure S20.** UV spectrum of isoamethinol B (2).

**Figure S21.** ^1^H NMR (600 MHz, CDCl_3_) spectrum of isoamethinol C (3).

**Figure S22.** ^13^C NMR (150 MHz, CDCl_3_) spectrum of isoamethinol C (3).

**Figure S23.** DEPT spectrum of isoamethinol C (3).

**Figure S24.** HSQC spectrum of isoamethinol C (3).

**Figure S25.** HMBC spectrum of isoamethinol C (3).

**Figure S26.** ^1^H-^1^H COSY spectrum of isoamethinol C (3).

**Figure S27.** NOESY spectrum of isoamethinol C (3).

**Figure S28.** IR spectrum of isoamethinol C (3).

**Figure S29.** HR-ESI-MS spectrum of isoamethinol C (3).

**Figure S30.** ^1^H NMR (600 MHz, CDCl_3_) spectrum of isoamethinol D (4).

**Figure S31.** ^13^C NMR (150 MHz, CDCl_3_) spectrum of isoamethinol D (4).

**Figure S32.** DEPT spectrum of isoamethinol D (4).

**Figure S33.** HSQC spectrum of isoamethinol D (4).

**Figure S34.** HMBC spectrum of isoamethinol D (4).

**Figure S35.** ^1^H-^1^H COSY spectrum of isoamethinol D (4).

**Figure S36.** NOESY spectrum of isoamethinol D (4).

**Figure S37.** IR spectrum of isoamethinol D (4).

**Figure S38.** HR-ESI-MS spectrum of isoamethinol D (4).

**Figure S39.** UV spectrum of isoamethinol D (4).

**Figure S40.** ^1^H NMR (600 MHz, CDCl_3_) spectrum of isoamethinol E (5).

**Figure S41.** ^13^C NMR (150 MHz, CDCl_3_) spectrum of isoamethinol E (5).

**Figure S42.** DEPT spectrum of isoamethinol E (5).

**Figure S43.** HSQC spectrum of isoamethinol E (5).

**Figure S44.** HMBC spectrum of isoamethinol E (5).

**Figure S45.** ^1^H-^1^H COSY spectrum of isoamethinol E (5).

**Figure S46.** NOESY spectrum of isoamethinol E (5).

**Figure S47.** IR spectrum of isoamethinol E (5).

**Figure S48.** HR-ESI-MS spectrum of isoamethinol E (5).

**Figure S49.** UV spectrum of isoamethinol E (5).

**Figure S50.** ^1^H NMR spectrum of compound 6.

**Figure S51.** ^13^C NMR spectrum of compound 6.

 **Figure S1.** ^1^H NMR spectrum (600 MHz, CDCl_3_) of isoamethinol A (1).

**Figure S2.** ^13^C NMR (150 MHz, CDCl_3_) spectrum of isoamethinol A (1).

**Figure S3.** DEPT-135 spectrum (*θ =* 135°) of isoamethinol A (1).

**Figure S4.** HSQC spectrum of isoamethinol A (1).

**Figure S5.** HMBC spectrum of isoamethinol A (1).

**Figure S6.** ^1^H–^1^H COSY spectrum of isoamethinol A (1).

**Figure S7.** NOESY spectrum of isoamethinol A (1).


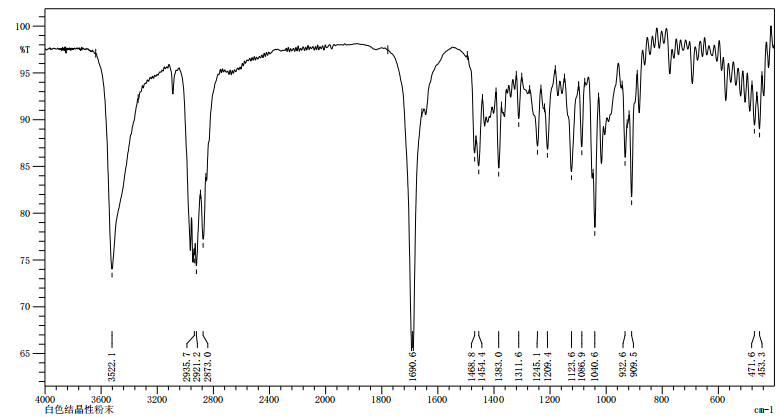


**Figure S8.** IR spectrum of isoamethinol A (1).

**Figure S9.** HR-ESI-MS spectrum of isoamethinol A (1).


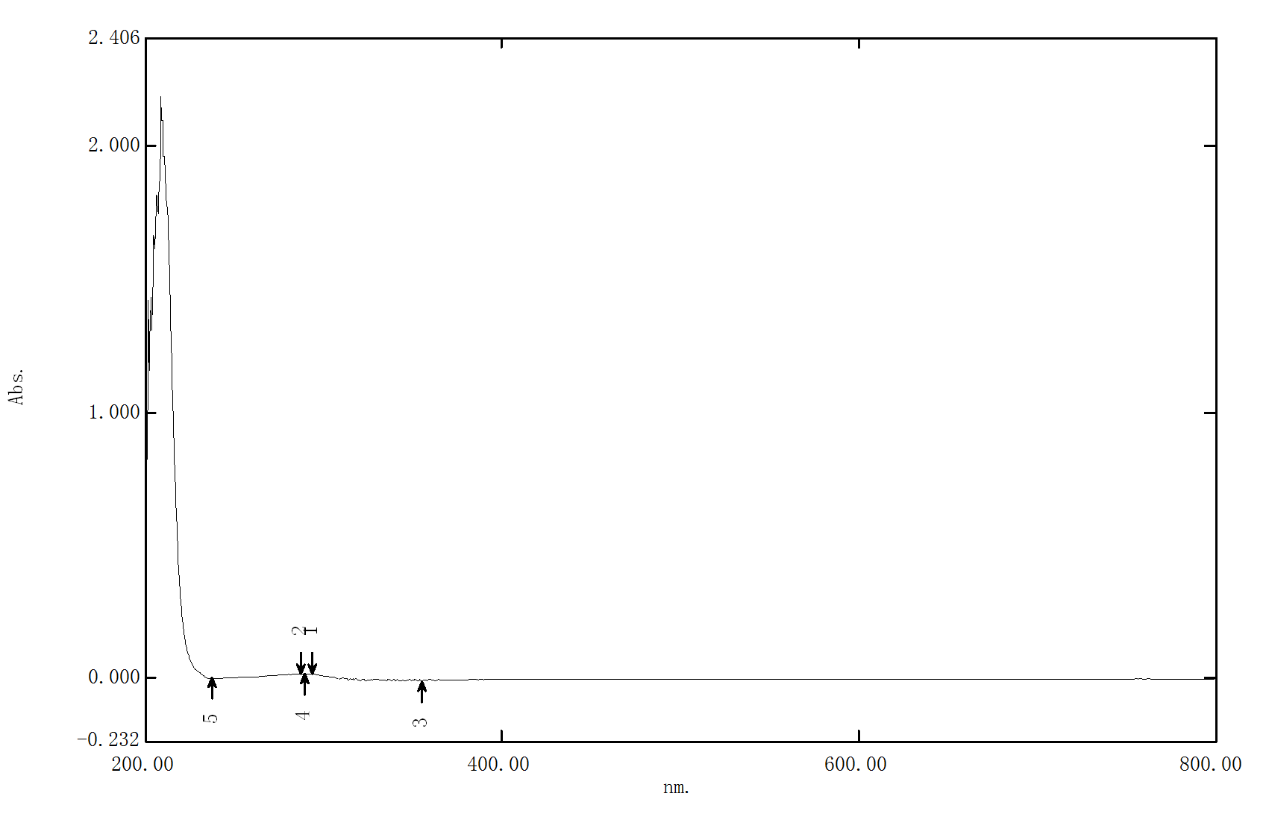


**Figure S10.** UV spectrum of isoamethinol A (1).

**Figure S11.** ^1^H NMR spectrum (400 MHz, CDCl_3_) of isoamethinol B (2).

**Figure S12.** ^13^C NMR (100 MHz, CDCl_3_) spectrum of isoamethinol B (2).

**Figure S13.** DEPT-135 spectrum (*θ =* 90°) of isoamethinol B (2).

**Figure S14.** HSQC spectrum of isoamethinol B (2).

**Figure S15.** HMBC spectrum of isoamethinol B (2).

**Figure S16.** ^1^H–^1^H COSY spectrum of isoamethinol B (2).

**Figure S17.** NOESY spectrum of isoamethinol B (2).


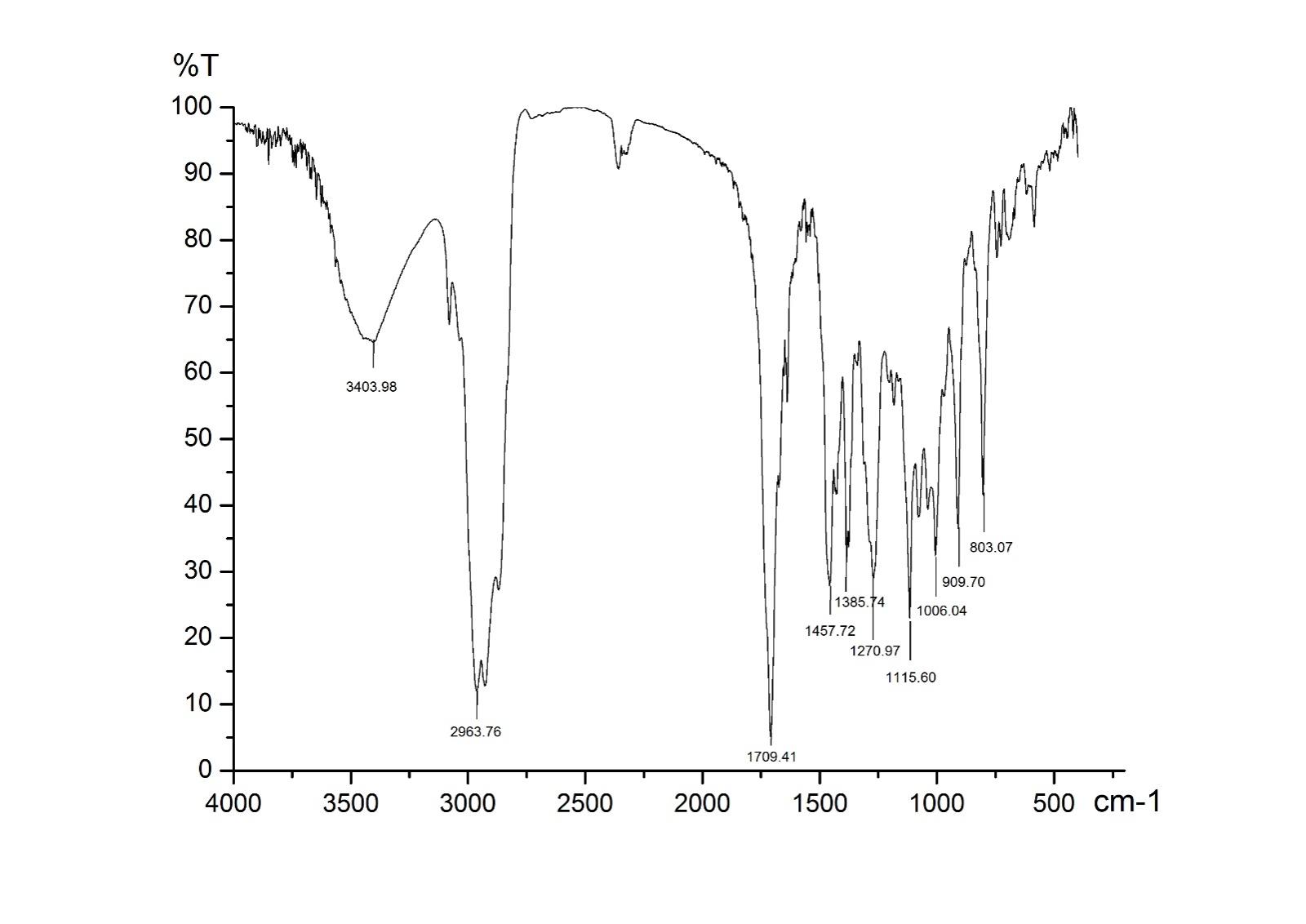


**Figure S18.** IR spectrum of isoamethinol B (2).

**Figure S19.** HR-ESI-MS spectrum of isoamethinol B (2).


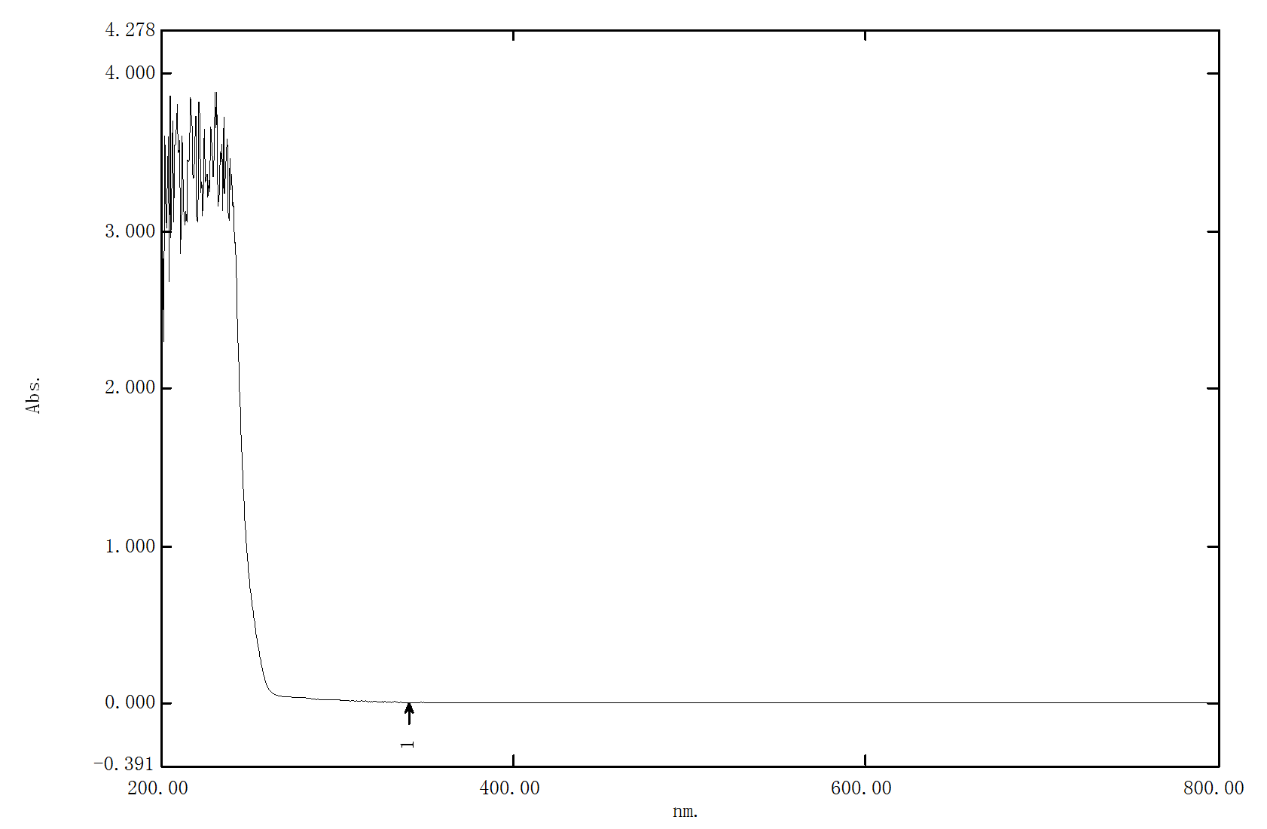


**Figure S20.** UV spectrum of isoamethinol B (2).

**Figure S21.** ^1^H NMR spectrum (600 MHz, CDCl_3_) of isoamethinol C (3).

**Figure S22.** ^13^C NMR spectrum (150 MHz, CDCl_3_) of isoamethinol C (3).

**Figure S23.** DEPT-135 spectrum (*θ =* 135°) of isoamethinol C (3).

**Figure S24.** HSQC spectrum of isoamethinol C (3).

**Figure S25.** HMBC spectrum of isoamethinol C (3).

**Figure S26.** ^1^H-^1^H COSY spectrum of isoamethinol C (3).

**Figure S27.** NOSEY spectrum of isoamethinol C (3).


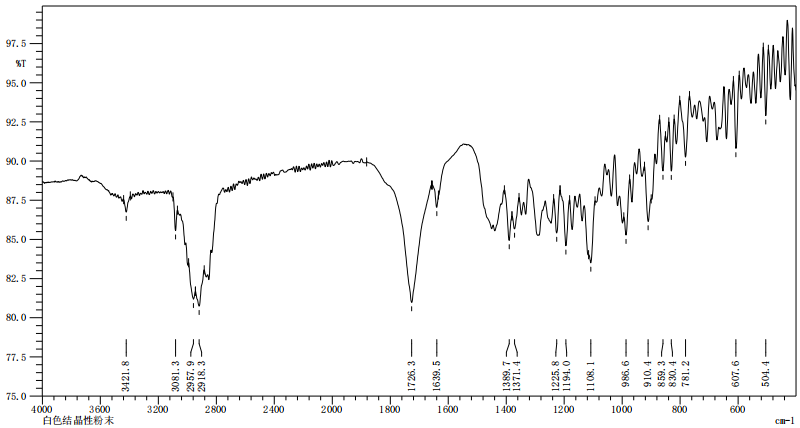


**Figure S28.** IR spectrum of isoamethinol C (3).

**Figure S29.** HR-ESI-MS spectrum of isoamethinol C (3).

**Figure S30.** ^1^H NMR spectrum (600 MHz, CDCl_3_) of isoamethinol D (4).

**Figure S31.** ^13^C NMR spectrum (150 MHz, CDCl_3_) of isoamethinol D (4).

**Figure S32.** DEPT-135 spectrum (*θ =* 135°) of isoamethinol D (4).

**Figure S33.** HSQC spectrum of isoamethinol D (4).

**Figure S34.** HMBC spectrum of isoamethinol D (4).

**Figure S35.** ^1^H-^1^H COSY spectrum of isoamethinol D (4).

**Figure S36.** NOESY spectrum of isoamethinol D (4).


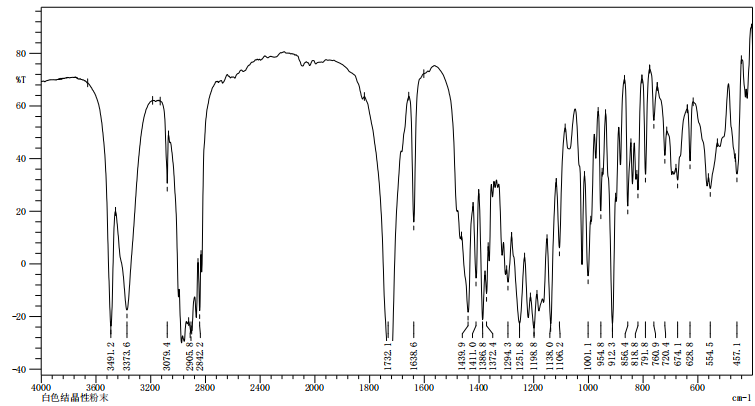


**Figure S37.** IR spectrum of isoamethinol D (4).

**Figure S38.** HR-ESI-MS spectrum of isoamethinol D (4).


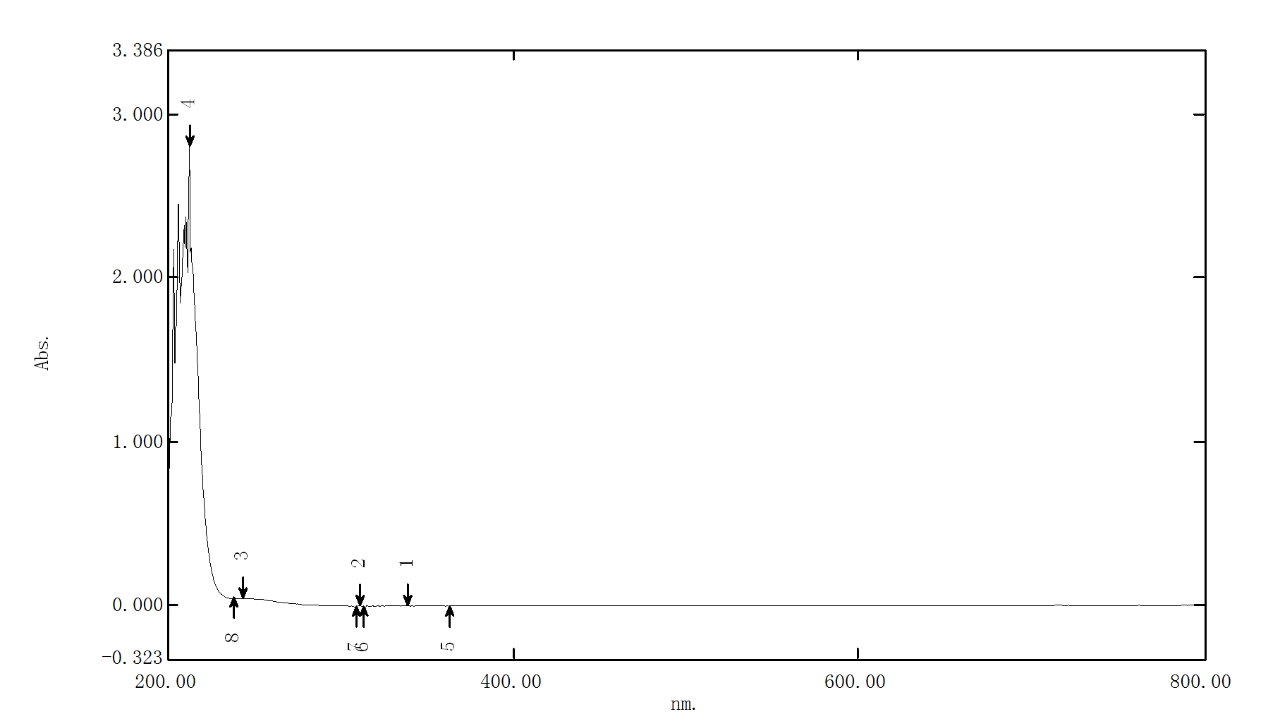


**Figure S39.** UV spectrum of isoamethinol D (4).

**Figure S40.** ^1^H NMR spectrum (600 MHz, CDCl_3_) of isoamethinol E (5).

**Figure S41.** ^13^C NMR spectrum (150 MHz, CDCl_3_) of isoamethinol E (5).

**Figure S42.** DEPT-135 spectrum (*θ =* 135°) of isoamethinol E (5).

**Figure S43.** HSQC spectrum of isoamethinol E (5).

**Figure S44.** HMBC spectrum of isoamethinol E (5).

**Figure S45.** ^1^H-^1^H COSY spectrum of isoamethinol E (5).

**Figure S46.** NOESY spectrum of isoamethinol E (5).


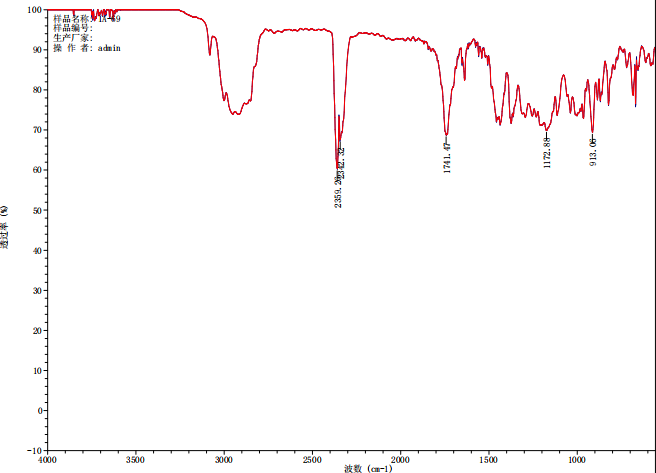


**Figure S47.** IR spectrum of isoamethinol E (5).

**Figure S48.** HR-ESI-MS spectrum of isoamethinol E (5).


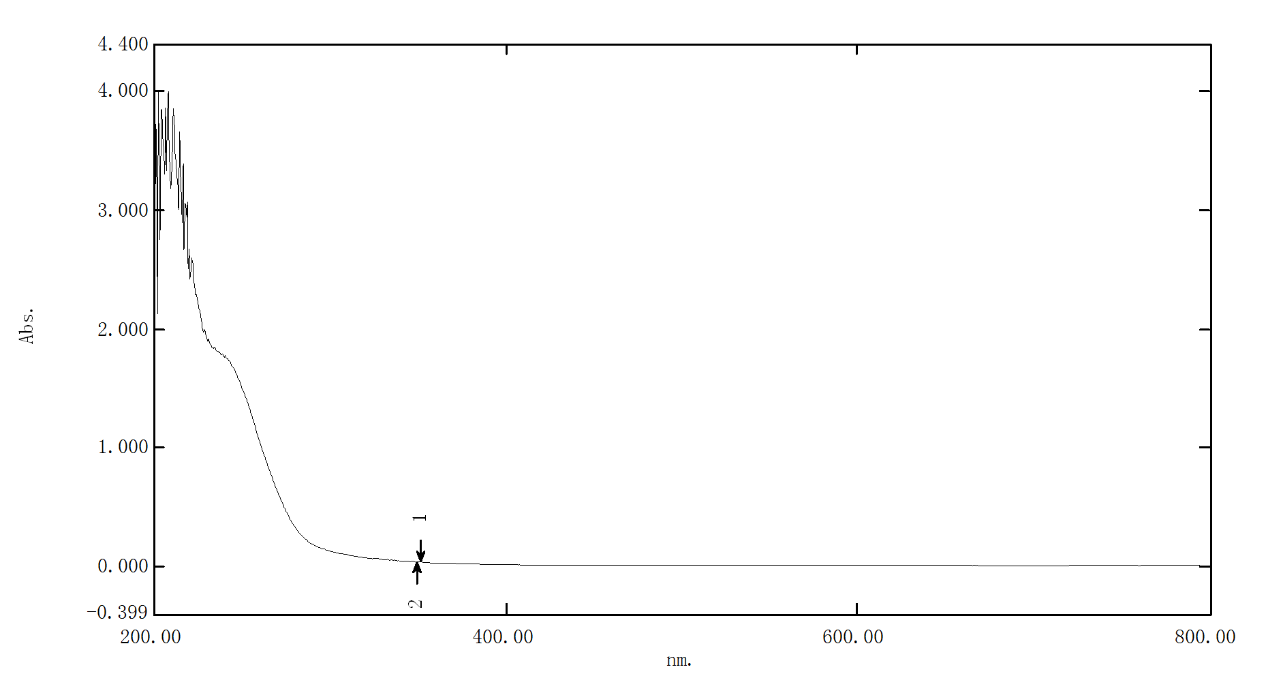


**Figure S49.** UV spectrum of isoamethinol E (5).

**Figure S50.** ^1^H NMR spectrum of compound 6.

**Figure S51.** ^13^C NMR spectrum of compound 6.
